# Impact of Bulk-Phase Self-Assembly on Growth and Activation of Aqueous Surfactant Aerosol

**DOI:** 10.1021/acs.est.4c11584

**Published:** 2025-07-10

**Authors:** Sampo Vepsäläinen, Nønne L. Prisle

**Affiliations:** † Nano and Molecular Systems Research Unit, 6370 University of Oulu, P.O. Box 3000, FI-90014 Oulu, Finland; ‡ Center for Atmospheric Research, 6370 University of Oulu, P.O. Box 4500, FI-90014 Oulu, Finland; § Center for Molecular Water Science, Deutsches Elektronen-Synchrotron DESY, Notkestrasse 85, D-22607 Hamburg, Germany; ∥ Institute of Inorganic and Applied Chemistry, University of Hamburg, Martin-Luther-King-Platz 6, D-20146 Hamburg, Germany

**Keywords:** atmospheric aerosol, surfactants, self-assembly, micelles, clusters, CCN activation, thermodynamic modeling

## Abstract

Surface active compounds (surfactants) have been found in atmospheric aerosols from many environments. In microscopic aqueous droplets, their presence may cause a variety of effects that are challenging to model comprehensively. In this work, we investigate the solute effects of atmospheric surfactant self-assembly to form micelles and small clusters in aqueous droplets. Several water activity models are employed in combination with Köhler theory to represent self-assembly phenomena within the droplet bulk while also accounting for surfactant bulk–surface partitioning. Our results show that surfactant self-assembly has only minor effects at the critical point of cloud droplet activation; however, very significant effects are observed during earlier stages of droplet growth at subsaturated ambient humidities. A major driver is the binding of free sodium counterions to the surfactant aggregates, strongly limiting the effect of dissolved sodium ions on droplet water activity. Variations in droplet equilibrium size at a given subsaturated humidity due to the presence of surfactant aggregates lead to different estimates of droplet water uptake and aerosol liquid water content. As a result, predictions of atmospheric processes involving surfactant aerosol could be highly sensitive to how surfactant self-assembly and related phenomena are taken into account.

## Graphical abstract

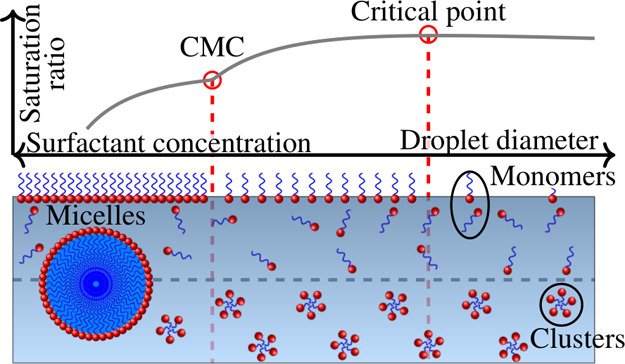

## 1 Introduction

Atmospheric aerosols remain one of the largest sources of uncertainty in estimating global radiative forcing.^1,2^ Surface active organic species (surfactants) are commonly present in atmospheric aerosol.^3–16^ Surfactants are typically amphiphiles, consisting of hydrophobic and hydrophilic parts. In aqueous solutions, they adsorb at the solution surface, where weaker intermolecular forces between many organic surfactants compared to between water molecules result in lower surface tension than that of pure water. The volume of atmospheric aqueous droplets is usually sufficiently small that the surfactant mass is significantly redistributed between the bulk and surface phases due to surface adsorption, a process called bulk–surface *partitioning*.^17–21^ Surfactants and surface tension can affect the critical point of cloud droplet activation of atmospheric aerosols, but the magnitudes of specific mechanisms are still not fully constrained.^18,20–23^

Some surfactants furthermore have the ability to self-assemble in the bulk phase of concentrated aqueous solutions, above a compound-specific limit referred to as the critical micelle concentration (CMC). The structures of the aggregates formed are governed by the balance of intermolecular forces^24^ and may be spherical or tubular micelles, lamellar phases, vesicles, or others.^25–28^ Investigations on the presence and importance of surfactant self-assembly in atmospheric aerosols have mainly focused on fatty acids.^28–31^ While surface active fatty acids and their deprotonated anions can self-assemble at very low concentrations,^29^ Köhler calculations have shown that the concentrations of fatty acid salts are below the respective CMCs at the point of cloud droplet activation.^17,32^ However, during earlier stages of droplet growth, where solute concentrations are much higher, the CMC may still be well exceeded.

Gérard et al.^10^ found surfactants to be present in all PM_2.5_ aerosols sampled from the Baltic Coast at Askö, Sweden. The concentrations varied between 27 ± 6 and 143 ± 29 mM within the aerosol phase and 104 ± 21 and 785 ± 157 pmol m^–3^ of ambient air. These surfactant concentrations were found to be sufficient to strongly depress the surface tension of atmospheric particles until the point of cloud droplet activation. Gérard et al.^16^ investigated PM_1_ aerosols from urban, coastal, and remote regions of Europe (Lyon, France, Rogoznica, Croatia, and Pallas, Finland, respectively) and found amphiphilic surfactants in concentrations up to 2.8 μg m^–3^ of ambient air and 1.3 M in the particle dry volume. They also obtained low CMC values (3 × 10^–5^–9 × 10^–3^ M) for these surfactant mixtures. This supports that the CMC can be exceeded at lower relative humidities, corresponding to droplet states during a major part of the droplet growth curve before activation. Aqueous droplets may also repeatedly shrink and grow during cloud processing in the atmosphere,^33^ causing variations in the water content that could lead to periodically exceeding the CMC.

Kirpes et al.^34^ found 53% of 300 individual Alaskan Arctic winter sea spray aerosol (SSA) particles, analyzed by Raman microspectroscopy, to contain at least one fatty acid. Cochran et al.^9^ tentatively identified over 280 organic compounds in freshly emitted SSA, including fatty acids and derivatives of fatty acids. The particles that constitute the peak of the nascent SSA number size distribution have dry diameters of approximately 0.1–0.2 μm.^35^ More than half of these particles is comprised by organic matter and the organic fraction has been found to increase with decreasing particle size.^35^ Zhao et al.^36^ determined the average concentration of total suspended particle mass from residential cooking alone as 138 μg/m^3^ of air in urban Guangzhou, China. Organic matter was the major component (66.9% by mass) and over 90 organic species were identified and quantified, explaining 14.5% of the organic compounds present. Fatty acids alone accounted for 75.7% of the total mass of the quantified organic compounds. Fatty acids have also been found to be significant in other urban environments^37,38^ and even to be one of the dominant organic classes in PM_2.5_ emissions from typical restaurants in Portugal.^38^

Pfrang et al.^28^ demonstrated that surfactant self-assembly into complex 3D aggregate structures occurs during dehumidification experiments of aqueous oleic acid–sodium oleate droplets with radii of 30 μm to 1 mm for humidity conditions relevant to the atmosphere (RH = 80–95%). The structures identified by Pfrang et al.^28^ are anisotropic and could therefore significantly affect the properties of atmospheric droplets and how they interact with light. However, droplets larger than 10 μm have very short atmospheric lifetimes due to gravitational settling.^39^ In smaller droplets with longer lifetimes, self-assembly phenomena may not impact the droplet properties in the same way as in larger droplets. Smaller droplets have larger surface-area-to-bulk-volume ratios and may be more bulk-depleted at the same surfactant concentration, such that the CMC is less likely to be exceeded than in larger droplets.^17,23^ However, the work of Pfrang et al.^28^ suggests that the potential presence of complex surfactant aggregate structures in atmospheric aerosols could affect key aerosol properties and should be considered.

Traditionally, aggregate structures formed above the CMC are thought to coexist in solution with the remaining surfactant monomers.^25,26,40^ However, recent work using relaxation and diffusion nuclear magnetic resonance (NMR) techniques with relaxation modeling suggests that surfactants may form various clusters, each consisting of a few monomers^41,42^ even below the CMC. Above the CMC, sizes of fatty acid micelles have conventionally been estimated based on NMR experiments in combination with the Stokes–Einstein equation to be in the range of 200 ± 100 nm.^42–44^ This size range is comparable to the size of entire atmospheric droplets. The presence of such micelles in micrometer-sized or smaller droplets relevant for the atmosphere is therefore not immediately evident, at least not analogously to macroscopic bulk phases. Other works have found micelles to be aggregates of roughly some tens to 100 monomers.^40,43,45,46^ With a footprint for a decanoate anion of 40.1 Å^2^,^47^ a simple estimate for the diameter of a sphere with surface area corresponding to the total footprint of this number of decanoate monomers is just a few nanometers, or 1–2 orders of magnitude smaller. This is consistent with recent relaxation modeling^41,42^ using parameters from molecular dynamics simulations estimating sodium decanoate micelles to comprise 48 monomers and be approximately 5.5 nm in diameter. Micelles in this size range could readily be contained within the bulk-phase of even submicron sized droplets and thus contribute to alter the properties of such droplets as they grow.

Calculations of cloud droplet growth and activation often make simplifying assumptions regarding the effects of micelles and surfactant CMC in the droplet phase. Typically, the surface tension at the CMC is imposed as a lower limit on the droplet surface tension, but the formation and presence of micelles is not explicitly addressed.^17,21,32,48^ Other droplet model studies have circumvented considerations of the CMC altogether by assuming that all surfactant in a droplet reside in an insoluble surface film.^49,50^ To the best of our knowledge, the presence and effects of micelles and other self-assembled structures themselves have not previously been taken explicitly into account in predictive Köhler calculations. Here, we investigate in detail the various solute effects of self-assembly during growth and activation of aqueous droplets comprising sodium decanoate, a relatively well understood surfactant which has been used as model for atmospheric surfactant aerosol in a number of previous works.^17,32,49,51^ Our aim is to investigate the potential impact of self-assembly phenomena, and the role of different processes involved, on the state and climate relevant properties of aqueous surfactant aerosol.

## 2 Theory and Modeling

We perform Köhler calculations for the hygroscopic growth and cloud droplet activation of sodium decanoate (hereafter denoted NaC_10_) particles with a range of initial dry particle sizes, while including the effects of surfactant self-assembly in the aqueous droplet bulk. The compound properties of pure water and NaC_10_ used in the calculations are summarized in Table 1. We employ several water activity models, described below, that each include different phenomena related to surfactant self-assembly, to assess the impact of each of these mechanisms in aqueous aerosol droplets. A conceptual representation of the modeling approach and different water activity models is shown in Figure 1. Throughout droplet growth, bulk–surface partitioning is accounted for using the surface Monolayer (ML) model of Malila and Prisle.^48^ These calculations are identical for all the different water activity models. From the predicted Köhler curves, we evaluate the hygroscopic growth factor (HGF) and aerosol liquid water content (LWC) corresponding to different stages of droplet growth to assess the impact of surfactant self-assembly via the droplet water activity evaluated for these properties.

**Table 1:** Molar Masses (*M*), Densities (ρ), and Surface Tensions (σ) of the Different Pure Compounds at 298.15 K

compound	*M* (g mol^–1^)	ρ (kg m^–3^)	σ (mN m^–1^)
water	18.0153	997.0^a^	72.0^b^
sodium decanoate (NaC_10_)	194.2470	1099.2^c^	22.5^d^

a Pátek et al.^54^

b IAPWS.^55^

c Extended from the binary density of water–NaC_10_ calculated via the method of Calderón and Prisle.^53^

d The value from the aqueous surface tension function at the CMC (see eq S9 of the Supplement).

**Figure 1:**
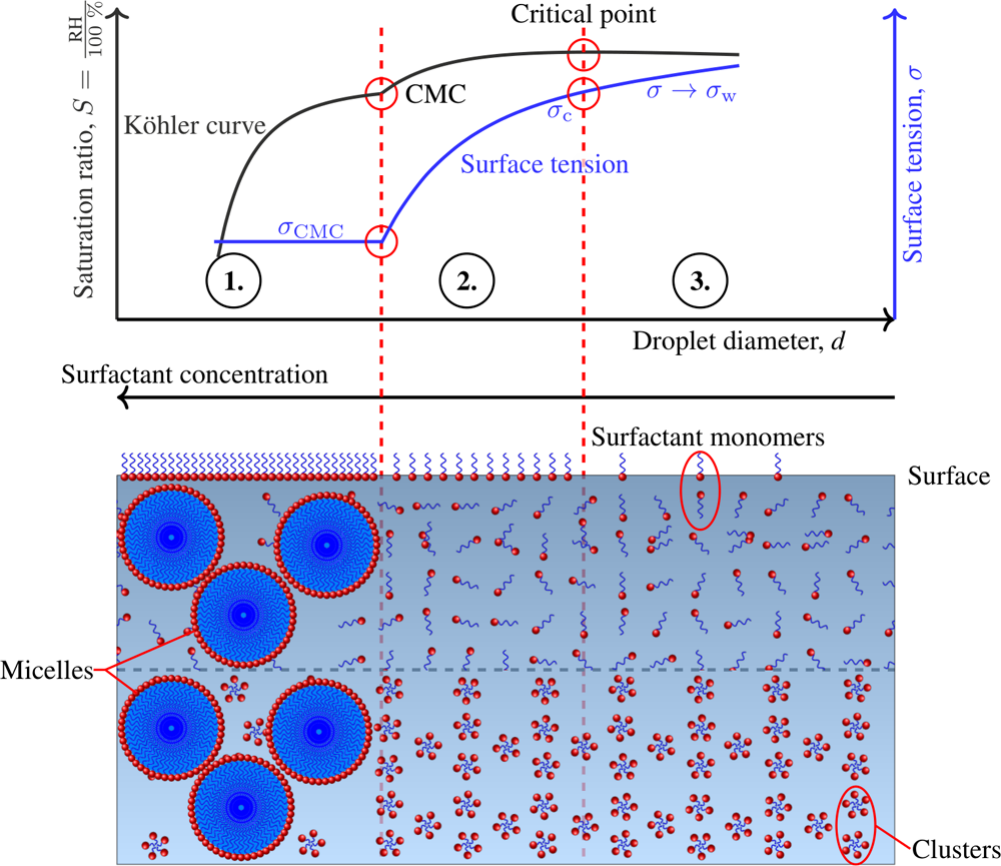
Conceptual overview of different water activity models and a resulting Köhler curve divided into three regions: **(1)** At lower relative humidities (RH), the surfactant concentration in the droplet bulk exceeds the CMC and micelles coexist in solution with surfactant monomers (MonoMic, CISA) or clusters (CluMic, CluMicNa). The surface phase is fully saturated with surfactant monomers, and the droplet surface tension remains constant at σ_CMC_. **(2)** As the growing droplet approaches the critical point of cloud droplet activation, the surfactant concentration in the droplet bulk dilutes below the CMC, and micelles are no longer present. Surfactant content in the droplet bulk phase is either monomers (Mono, MonoMic, CISA) or clusters (Clu, CluMic, CluMicNa). The droplet surface tension increases and reaches the value σ_c_ at the critical point. **(3)** The critical point has been exceeded, and the droplets are activated. Surfactant content in the droplet bulk phase remains similar to region **(2)**. The surface tension changes more slowly than in region **(2)**, approaching that of pure water (σ_w_).

### 2.1 Köhler Theory

Cloud droplets in the atmosphere form when water condenses onto the surfaces of aerosol particles. The process of droplet growth and activation into cloud droplets is described by the Köhler equation,^32,52^ which gives the equilibrium water vapor saturation ratio *S* over a spherical droplet with diameter *d*

$$S≡\frac{p_{w}}{p_{w}^{0}}=a_{w}\exp\left(\frac{4M_{w}\sigma }{RT\rho_{w}d}\right)=\frac{\mathrm{RH}}{100\%}$$ 1

Here, *p*_w_ is the equilibrium partial pressure of water over the aqueous droplet, *p*_w_^0^ is the saturation vapor pressure over a flat surface of pure water, *a*_w_ is the droplet solution water activity (Raoult term), *M*_w_ and ρ_w_ are the molar mass and density of water (Table 1), σ is the droplet surface tension, *R* is the universal gas constant, *T* is the temperature in Kelvin, and RH is the relative humidity. The critical point of cloud droplet activation is determined as the critical saturation ratio (*S*_c_), or the critical supersaturation SS_c_ = (*S*_c_ – 1) × 100%, corresponding to the maximum value of the Köhler curve described by eq 1 and the corresponding droplet diameter *d*_c_.

The overall composition of a growing droplet is determined from the initial total number of solute monomers (*n*_NaC_10__^T^) contained in a spherical dry particle with a diameter *D*_p_ and the number of condensed water molecules. The total number of solute monomers in the droplet remains constant while it grows by condensation of water vapor. In a droplet of a given diameter *d*, the total number of water molecules (*n*_w_^T^) is determined iteratively from

$$\frac{\pi }{6}d^{3}\rho \left(x^{T}\right)=n_{{\mathrm{NaC}}_{10}}^{T}m_{{\mathrm{NaC}}_{10}}+n_{w}^{T}m_{w}$$ 2

where ρ­(**x** ^T^) is the droplet density^53^ as a function of the droplet composition in terms of the mole fraction vector **x** ^T^ = (*x*_w_^T^, *x*_NaC_10__^T^), and *m*_*j*_ = *M*_*j*_/*N*_A_ is the mass of component *j*, where *M*_*j*_ is the molar mass of component *j* (Table 1) and *N*_A_ is Avogadro’s constant.

### 2.2 Molecular Monolayer Bulk–Surface Partitioning Model

Our calculations account for bulk–surface partitioning of droplet components during growth and activation with the molecular Monolayer (ML) model of Malila and Prisle,^48^ which is used to determine the composition of the droplet bulk and surface phases. The Monolayer model has previously been successfully used for a variety of aqueous surfactant droplet systems and conditions.^18,20,21,23,48,56,57^ The model divides the spherical aqueous droplet into a surface monolayer of thickness δ and an interior bulk solution with diameter *d* – 2δ and calculates the full compositions of both the droplet bulk and surface phases. The surface is described as a liquid phase with a composition distinct from the bulk. The partitioning between the bulk and surface phases for each compound *j* in the droplet is calculated iteratively by relating the droplet surface tension σ, as a function of bulk composition **x** ^B^ = (*x*_w_^B^, *x*_NaC_10__^B^), to the surface composition **x** ^S^ = (*x*_w_^S^, *x*_NaC_10__^S^) as

$$\sigma \left(x^{B},T\right)=\frac{\underset{j}{\sum }\sigma_{j}v_{j}x_{j}^{S}}{\underset{j}{\sum }v_{j}x_{j}^{S}}$$ 3

where *x*_*j*_^S^ and *x*_*j*_^B^ are the droplet surface and bulk mole fractions of compound *j*, while *v*_*j*_ is the liquid phase molecular volume, and σ_*j*_ the pure component surface tension of compound *j*. The condition of mass conservation (*n*_*j*_^T^ = *n*_*j*_^S^ + *n*_*j*_^B^) is imposed on the calculations for each compound *j*. The thickness of the spherical shell that comprises the surface monolayer^48^ is calculated as

$$\delta ={\left(\frac{6}{\pi }\underset{j}{\sum }v_{j}x_{j}^{S}\right)}^{1/3}$$ 4

To preserve electrical neutrality of each phase, it is assumed that partitioning of surface active NaC_10_ to the droplet surface takes place for undissociated monomers.^17,21,48^ The surface partitioned undissociated salt monomers therefore do not contribute to the amount of free sodium ions, Na^+^, in the droplet bulk. When the concentration of surfactant monomers remaining in the droplet bulk after partitioning is below the CMC, the droplet surface tension is set equal to that of an equivalent macroscopic solution with the same bulk phase composition (see Supplement Section S3.1). If the surfactant concentration remaining in the droplet bulk exceeds the CMC, the surface tension is that at the CMC (σ_CMC_ = 22.5 mN m^–1^) which is assumed equal to the surface tension of the pure (subcooled liquid) surfactant.^18,23,48^

### 2.3 Water Activity Models

Surfactant self-assembly in the droplet bulk phase is taken into account with several models, each describing the effects of various phenomena on water activity (*a*_w_) in the Köhler equation (eq 1). In most of the models, we approximate the droplet water activity as a corrected water mole fraction *x*_w_^B^ with different assumptions regarding the dissolution and aggregation state of NaC_10_ in the bulk phase. We also use a comprehensive thermodynamic activity model^40^ to evaluate the water activity of growing aqueous sodium decanoate droplets. The number of NaC_10_ monomers in the bulk phase *n*_NaC_10__^B^ and the droplet surface tension σ at a given droplet size *d* are identical for all water activity calculations. To calculate *a*_w_, each surfactant monomer in the droplet bulk, including those contained in aggregates, are assumed to be fully dissociated in solution

$$\mathrm{Na}C_{10}\to {\mathrm{Na}}^{+}+C_{10}^{-}$$ 5

where C_10_^–^ refers to the deprotonated decanoate anion (C_10_H_19_O_2_^–^). The combination of different water activity models with the Köhler eq 1 is illustrated in Figure 1.

#### 2.3.1 Dissolved Sodium Decanoate Monomers

In the simplest water activity model (Mono), all NaC_10_ in the droplet bulk is assumed to exist as dissociated monomers at concentrations up to the CMC. Above the CMC, the droplet bulk is assumed to contain an amount of dissolved NaC_10_ monomers equal to the CMC, *n*_NaC_10__^CMC^. The maximum amount of dissolved NaC_10_ increases as the droplet volume grows with increasing *d* until all NaC_10_ in the bulk phase is dissolved. Excess NaC_10_ above the CMC remains undissolved and has negligible impact on the droplet volume and solution state, resulting in a constant water activity above the CMC. The water activity (*a*_w_) is calculated as

$$a_{w}=\frac{n_{w}^{B}}{n_{w}^{B}+n_{C_{10}^{-}}^{B}+n_{{\mathrm{Na}}^{+}}^{B}}$$ 6

where *n*_Na^+^_^B^ = *n*_C_10_^–^_^B^ = *n*_NaC_10__^B^ ≤ *n*_NaC_10__^CMC^. The inequality denotes that the maximum number of dissociated NaC_10_ monomers in the droplet bulk is limited by the CMC.

#### 2.3.2 Monomers Coexist with Micelles

In the second model (MonoMic), the amount of NaC_10_ exceeding the CMC is assumed to form micelles (mic) coexisting with the monomers in the droplet bulk. Each micelle consists of *n*_m_ = 48 C_10_^–^ anions^42^ and the aggregation number *n*_m_ is assumed to be independent of the NaC_10_ concentration and therefore constant during droplet growth. The water activity is calculated as

$$a_{w}=\frac{n_{w}^{B}}{n_{w}^{B}+n_{C_{10}^{-}}^{B}+n_{\mathrm{mic}}^{B}+n_{{\mathrm{Na}}^{+}}^{B}}$$ 7

where *n*_mic_^B^ is the number of micelles in the droplet bulk, estimated from the amount of NaC_10_ left in the bulk phase with the fraction of micellization (Section S3.4 of the Supplement), and *n*_Na^+^_^B^ = *n*_C_10_^–^_^B^ + *n*_*m*_·*n*_mic_^B^, with *n*_C_10_^–^_^B^ ≤ *n*_NaC_10__^CMC^.

#### 2.3.3 Formation of Small Clusters

Recent work suggests that NaC_10_ does not exist purely as monomers below the CMC, but instead can form small clusters.^42^ In the third model (Clu), we therefore assume all NaC_10_ in the droplet bulk to form clusters (clu), each consisting of a constant *n*_c_ = 5 C_10_^–^ anions.^42^ Similarly to the Mono model, excess NaC_10_ above the CMC is assumed to remain undissolved and have negligible impact on the droplet volume and the solution state. The water activity is calculated as

$$a_{w}=\frac{n_{w}^{B}}{n_{w}^{B}+n_{\mathrm{clu}}^{B}+n_{{\mathrm{Na}}^{+}}^{B}}$$ 8

where *n*_clu_^B^ is the number of clusters in the droplet bulk, and *n*_Na^+^_^B^ = *n*_clu_^B^·*n*_c_ ≤ *n*_NaC_10__^CMC^.

#### 2.3.4 Clusters Coexist with Micelles

In the fourth model (CluMic), all NaC_10_ monomers in the droplet bulk are assumed to form clusters (*n*_c_ = 5) below the CMC. Above the CMC, the clusters coexist with micelles (*n*_m_ = 48). The water activity is calculated as

$$a_{w}=\frac{n_{w}^{B}}{n_{w}^{B}+n_{\mathrm{clu}}^{B}+n_{\mathrm{mic}}^{B}+n_{{\mathrm{Na}}^{+}}^{B}}$$ 9

where *n*_Na^+^_^B^ = *n*_clu_^B^·*n*_c_ + *n*_mic_^B^·*n*_m_ and *n*_clu_^B^·*n*_c_ ≤ *n*_NaC_10__^CMC^.

#### 2.3.5 Clusters Coexist with Micelles with Counterion Binding

In the fifth model (CluMicNa), we account for the electrostatic association of dissolved Na^+^ counterions to the decanoate anion clusters or micelles. This has been found to significantly limit the amount of free Na^+^ counterions in solution.^40,45,58,59^ We combine the CluMic model with a simple parametrization of the fraction of free sodium ions *f*_Na^+^_, based on linear interpolation of experimental observations using NMR.^42^ At NaC_10_ concentrations in the droplet bulk phase below 50 mM, *f*_Na^+^_ = 1, and for NaC_10_ concentrations above 700 mM, *f*_Na^+^_ = 0.41. The droplet water activity is calculated as

$$a_{w}=\frac{n_{w}^{B}}{n_{w}^{B}+n_{\mathrm{clu}}^{B}+n_{\mathrm{mic}}^{B}+f_{{\mathrm{Na}}^{+}}n_{{\mathrm{Na}}^{+}}^{B}}$$ 10

where, as for the CluMic model, *n*_Na^+^_^B^ = *n*_c_·*n*_clu_^B^ + *n*_m_·*n*_mic_^B^ = *n*_NaC_10__^B^ and *n*_c_·*n*_Clu_^B^ ≤ *n*_NaC_10__^CMC^.

#### 2.3.6 CMC-Based Ionic Surfactant Activity Model

In addition to the corrected water mole fraction models, we use the comprehensive thermodynamic *CMC based Ionic Surfactant Activity model (CISA)* introduced by Calderón et al.^40^ to calculate the water activity for growing droplets along the Köhler curves. The CISA model considers the dissociation and micellization of an ionic surfactant as well as counterion binding to the micelles. We consider micelles comprising *n*_m_ = 48 C_10_^–^ anions^42^ coexisting with dissolved NaC_10_ monomers. A constant fraction of 0.68 sodium counterions bound to each micelle is assumed.^45,60^ The possible presence of small clusters is not explicitly accounted for by CISA or its underlying theoretical framework, which predate their discovery. Further details on calculations using the CISA model are given in the Supplement Section S2.1.

### 2.4 Aerosol Water Uptake

We use the hygroscopic growth factor (HGF) to directly compare the effects of surfactant self-assembly on droplet growth under the same humidity conditions. The HGF is calculated as

$$\mathrm{HGF}(\mathrm{RH})=\frac{d(\mathrm{RH})}{D_{p}}$$ 11

where RH is the ambient relative humidity from eq 1 and *D*_p_ is the dry particle diameter. The aerosol liquid water content (LWC) in a volume of air containing *N* droplets is calculated as

$$\mathrm{LWC}=n_{w}^{T}m_{w}N$$ 12

where *n*_w_^T^ is the total number of water molecules in a droplet with diameter *d* at a given RH, obtained from eq 2, and *m*_w_ = *M*_w_/*N*_A_.

## 3 Results and Discussion

### 3.1 Köhler Curves

The Köhler equilibrium growth curves calculated from eq 1 with the Mono, MonoMic, CISA, Clu, CluMic, and CluMicNa water activity models are presented in Figure 2 for NaC_10_ particles with initial dry diameters *D*_p_ = 40 nm (a), 80 nm (b), 120 nm (c), and 160 nm (d), respectively. The critical supersaturations (SS_c_), droplet diameters (*d*_c_), and surface tensions (σ_c_) at the critical points of cloud droplet activation (marked as a point for each Köhler curve in Figure 2) are given in Table 2. Critical supersaturations denoted as SS_c_^exp^ (dashed horizontal lines) have been estimated by fitting power functions to experimental data measured with a static thermal gradient diffusion cloud condensation nucleus (CCN) counter by Prisle et al.^32^ (Section S3.5 of the Supplement). In each panel of Figure 2, the surface tension and bulk–surface partitioning equilibrium at a given droplet size is the same for calculations with all water activity models. The specific effects of surface tension and surfactant bulk–surface partitioning on Köhler predictions of cloud droplet growth and activation have been investigated extensively in previous works.^17,19,23,48,56,57,61,62^ We here focus on effects specifically introduced by different surfactant self-assembly phenomena in the droplet bulk phase.

**Table 2:** Droplet Diameter (*d*_c_), Supersaturation (SS_c_) and Surface Tension (σ_c_) at the Critical Point of Cloud Droplet Activation Corresponding to Figure 2 Predicted with Mono, MonoMic, CISA, Clu, CluMic, and CluMicNa at 298.15 K for Dry NaC_10_ Particles at *D*_p_ = 40, 80, 120, and 160 nm

Mono				MonoMic				CISA			
*D*_p_ (nm)	*d*_c_ (nm)	SS_c_ (%)	σ_c_ (mN m^–1^)	*D*_p_ (nm)	*d*_c_ (nm)	SS_c_ (%)	σ_c_ (mN m^–1^)	*D*_p_ (nm)	*d*_c_ (nm)	SS_c_ (%)	σ_c_ (mN m^–1^)
40.0	186.0	0.68	45.9	40.0	186.0	0.68	45.9	40.0	185.0	0.68	45.7
80.0	511.0	0.25	49.0	80.0	511.0	0.25	49.0	80.0	507.0	0.25	48.7
120.0	918.0	0.14	51.4	120.0	918.0	0.14	51.4	120.0	910.0	0.14	51.2
160.0	1388.0	0.1	53.3	160.0	1388.0	0.1	53.3	160.0	1378.0	0.1	53.1

Clu				CluMic				CluMicNa			
*D*_p_ (nm)	*d*_c_ (nm)	SS_c_ (%)	σ_c_ (mN m^–1^)	*D*_p_ (nm)	*d*_c_ (nm)	SS_c_ (%)	σ_c_ (mN m^–1^)	*D*_p_ (nm)	*d*_c_ (nm)	SS_c_ (%)	σ_c_ (mN m^–1^)
40.0	177.0	0.7	44.0	40.0	177.0	0.7	44.0	40.0	177.0	0.7	44.0
80.0	470.0	0.27	46.2	80.0	470.0	0.27	46.2	80.0	470.0	0.27	46.2
120.0	828.0	0.15	48.3	120.0	828.0	0.15	48.3	120.0	828.0	0.15	48.3
160.0	1237.0	0.1	50.0	160.0	1237.0	0.1	50.0	160.0	1237.0	0.1	50.0

**Figure 2:**
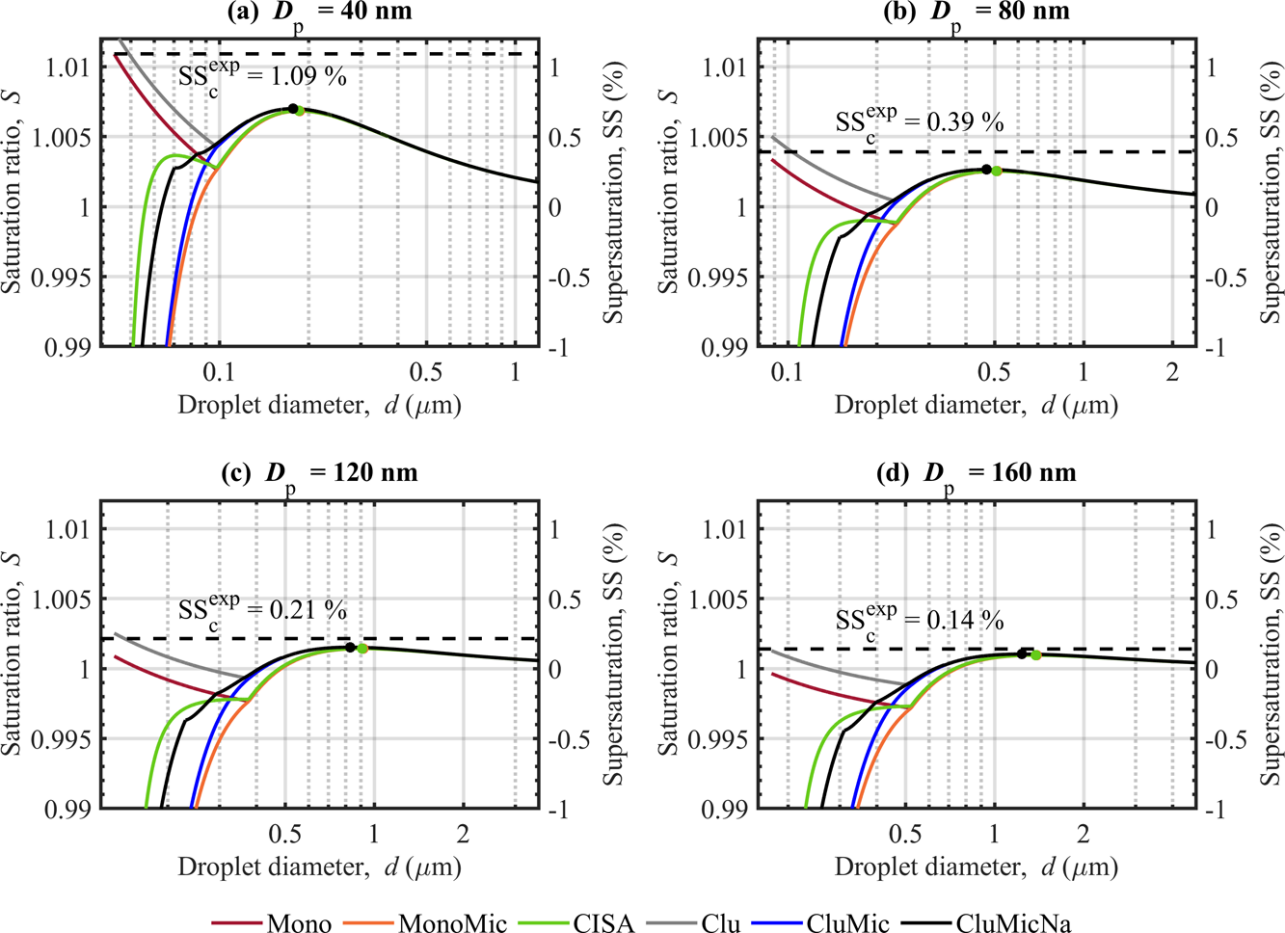
Köhler curves predicted with the Mono, MonoMic, CISA, Clu, CluMic, and CluMicNa water activity models for NaC_10_ particles with dry diameters of *D*_p_ = 40 nm (a), 80 nm (b), 120 nm (c), and 160 nm (d). The left *y*-axis indicates the saturation ratio $S=\frac{\mathrm{RH}}{100\%}$ from eq 1, and the right is the corresponding supersaturation SS = (*S* – 1) × 100%. The critical points of each Köhler curve are marked and also given in Table 2. Critical supersaturations SS_c_^exp^, calculated from a power function fitted to experimental CCN data by Prisle et al.^32^ (Section S3.5 of the Supplement), are shown as dashed black lines in each panel.

It is clear that surfactant self-assembly and possible counterion binding to the formed aggregates included in the different water activity models have only minor effects on predictions of SS_c_. This supports previous assessments that cloud droplet activation occurs for droplet solution states which are sufficiently dilute that the surfactant concentration does not exceed the CMC.^17,32^ Furthermore, droplet activation occurs at only slightly lower SS_c_, larger *d*_c_, and higher σ_c_ for the Mono, MonoMic, and CISA models (no clusters) than for the Clu, CluMic, and CluMicNa models. This shows that also effects caused by the formation of small surfactant clusters in the droplet bulk phase have only minor impact at the point of droplet activation (Table 2). The SS_c_ predicted with the different water activity models in this work are smaller than the experimental SS_c_^exp^ values of Prisle et al.,^32^ but the agreement improves as the particle size increases between panels. The Clu, CluMic, and CluMicNa models predict slightly higher SS_c_ that agree slightly better with SS_c_^exp^ than the Mono, MonoMic, or CISA models (no clusters), suggesting that small clusters could indeed be present in the bulk phase of activating droplets. However, the mutual differences between predictions that include cluster formation and those where NaC_10_ remains as monomers are minor. Direct experimental investigations would therefore be needed to firmly establish the presence of clusters in activating droplets.

Before the critical point, the shape of the Köhler curves varies significantly when different self-assembly phenomena in the bulk phase of growing droplets are accounted for. In particular, counterion binding to the micelles (CISA and CluMicNa models) leads to smaller equilibrium droplet sizes at a given RH, compared to considering micelle formation without counterion binding (MonoMic and CluMic models). The trends in Köhler predictions with the different water activity models are similar across the four dry particle sizes (*D*_p_) in Figure 2. In the region where NaC_10_ concentration exceeds the CMC (see Figure 1), saturation ratios predicted with the Mono and Clu models decrease exponentially with increasing droplet size. These two models do not consider micelle formation and the concentration of NaC_10_ in the droplet bulk is limited by the CMC. Above the CMC, any excess NaC_10_ in the bulk is assumed to not affect the water activity. This results in a constant *a*_w_ at small droplet sizes (Figure 3), such that the Kelvin term (exponential part of eq 1) dominates the corresponding part of the Köhler curves. This phenomenon is similar to that caused by limited aqueous bulk solubility of droplet components.^17,32,63^ The CISA and CluMicNa models predict inflection points before the critical point of cloud droplet activation, resulting from the effect of counterion binding on the water activity (Figure 3), in combination with the Kelvin effect. The inflection points are more prominent for smaller particles, due to the stronger Kelvin effect, and could stabilize growing droplets before activation, affecting the droplet–vapor interaction in a dynamic system, such as a rising air parcel.

**Figure 3:**
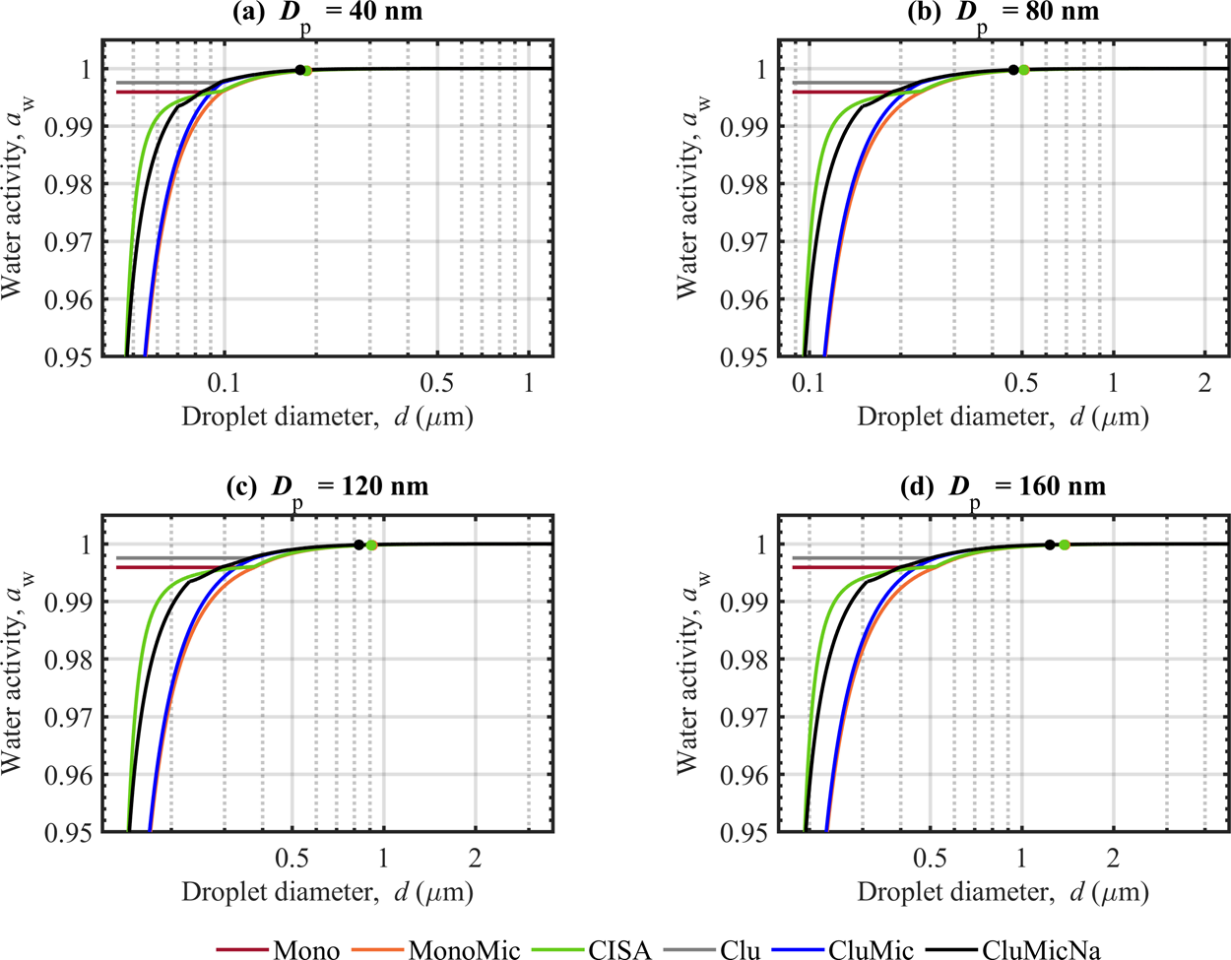
Droplet bulk-phase water activity evaluated along the Köhler curves with the Mono, MonoMic, CISA, Clu, CluMic, and CluMicNa water activity models for NaC_10_ particles of *D*_p_ = 40 nm (a), 80 nm (b), 120 nm (c), and 160 nm (d). Droplet sizes corresponding to the critical points of activation have been marked on each curve.

Once droplets are sufficiently dilute that the NaC_10_ bulk concentrations are below the CMC, the Mono, MonoMic, and CISA models (no clusters) predict similar Köhler curves. Analogously, the Köhler curves of the Clu, CluMic, and CluMicNa models agree well for droplet sizes where NaC_10_ bulk concentration is below the CMC. The close agreement between the models that consider counterion binding (CISA and CluMicNa) and those that do not (Mono, MonoMic, Clu, and CluMic) confirms that the effects of counterion binding are vanishing with increasing dilution as the growing droplet approaches activation and micelles are no longer present.

### 3.2 Droplet Water Activity

The bulk phase water activities for growing droplets corresponding to the Köhler curves in Figure 2 are shown in Figure 3. The presence of micelles significantly reduces water activity, but the CMC limits the amount of surfactant solute in the droplet bulk. Considering micelles (MonoMic, CluMic, CluMicNa, and CISA models) leads to significant reduction in water activity, compared to the Mono and Clu models (no micelles), primarily by allowing more dissolved sodium counterions in solution above the CMC. For example, between *D*_p_ = 40 and 160 nm (panels (a)–(d) in Figure 3), we find decreases in *a*_w_ of ∼10–12% for the MonoMic and CluMic models, and ∼4–5% for the CISA and CluMicNa models, each compared to the Mono model, at droplets sizes *d* = 1.2*D*_p_ (corresponding to HGF = 1.2, see eq 11). Including counterion binding to the micelles (CISA and CluMicNa models) limits the amount of free Na^+^ ions in the droplet bulk, leading to higher water activities compared to the MonoMic and CluMic models (no counterion binding). As a result, counterion binding shifts the Köhler curves (Figure 2) of the CluMicNa and CISA models to smaller equilibrium droplet sizes, compared to the MonoMic and CluMic models, when micelles are present in the droplets. The comprehensive thermodynamic model CISA overall agrees well with the simpler CluMicNa model (differences in *a*_w_ are <1% at HGF ≥1.2, for *a*_w_ > 0.95). This suggests that, among the simple water activity models used in this work, the combination of self-assembly effects considered in the CluMicNa model (both micelle formation and counterion binding) is most representative of the comprehensive thermodynamic CISA model.

The water activity models presented here consider simple micelle populations characterized by a single uniform aggregation number. Even the comprehensive thermodynamic CISA model^40^ considers only micelles and has been validated up to a surfactant molality of 1.6 mol kg^–1^ ∼ 1246 mM, significantly less than the surfactant bulk concentrations estimated in the present work at RH ∼ 95% (Figure S1 of the Supplement). Recent NMR experiments found evidence for presence of vesicles in aqueous solutions at very high NaC_10_ concentrations, several times above the CMC.^41,42^ In our calculations, such concentrations are realized at relative humidities below ∼99% (Figure S1 of the Supplement). During dehumidification experiments of aqueous oleic acid–sodium oleate droplets, evidence has been found for even more complex structures, including hexagonal close-packed inverted micelles, between RH = 80–95%.^28^ These more complex self-assembly phenomena could potentially also occur in atmospheric aerosol droplets, simultaneously with simpler micelles, in particular at high surfactant concentrations during early stages of droplet growth, corresponding to humidities far below saturation. However, this has not been established for realistic submicron droplet sizes and solute mixtures with existing experimental methods.

Atmospheric aerosols and droplets can comprise complex mixtures of several surfactant species^9,10,16^ and soluble organic and inorganic species, which may affect the interactions and nature of the self-assembly phenomena in the droplet bulk. For example, electrolytes could introduce high ionic strength in droplets at low humidities, which may affect both the CMC, the nature of self-assembled structures, and counterion binding in a solution. However, the magnitude and direction of such changes depend on the properties of interacting ions and typically remain poorly quantified across varying solution states.^24,40,53^

The present work employs thermodynamic models, which by definition assume that the system is at equilibrium and do not consider time-scales of aggregate formation or other kinetic solution effects. As the mean lifetime of an amphiphilic molecule in a small micelle is very short (10^–5^–10^–3^ s),^27^ we consider this to be a reasonable assumption for the present purposes. The simple corrected water mole fraction water activity models introduced here are compared to assess of the relative potential impact of small clusters, micelles, and counterion binding on equilibrium growth and activation of aqueous surfactant droplets. In future work, we recommend to extend considerations to include coexistence of both monomers and multiple self-assembled structures (small clusters, simple micelles, vesicles, etc.) in growing droplets. However, there are currently no comprehensive thermodynamic models to describe several complex self-assembly phenomena occurring simultaneously in aqueous droplets. Furthermore, lack of explicit data on the fraction and potential concentration dependence of small cluster formation, or other self-assembled structures coexisting with micelles, currently prohibit such considerations.

### 3.3 Particle Water Uptake

In Figure 4, we present the hygroscopic growth factors (HGF) calculated with eq 11 at RH = 95–99.9% for initially dry particles with sizes *D*_p_ = 30–200 nm, using the MonoMic, CISA, CluMic, and CluMicNa water activity models. The equilibrium droplet sizes *d* corresponding to a dry particle with size *D*_p_ at a given RH are obtained from the Köhler curves calculated with eq 1 for each water activity model. Figure 5 shows the aerosol liquid water content (LWC) calculated using eq 12 for a population of *N* = 1000 cm^–3^ droplets with each dry particle size *D*_p_, at humidities 99.9 and 95% RH, from predictions of the different water activity models. Figure S2 of the Supplement shows the LWC calculated for RH = 96–99%. HGF and LWC for the Mono and Clu models are not shown because they do not yield equilibrium curves at subsaturation for small dry particle sizes (see Figure 2). At larger particle sizes, the subsaturated Köhler curves for the Mono and Clu models show two coexisting equilibrium droplet sizes at the same RH. In each case, the larger of these corresponds to the MonoMic and CluMic models. For the smaller droplets, the solution state is unchanged below the sizes where the CMC is exceeded. This results in a constant *a*_w_ (Figure 3) and the Köhler curve is dominated by the Kelvin effect (Figure 2), as discussed in Section 3.1.

**Figure 4:**
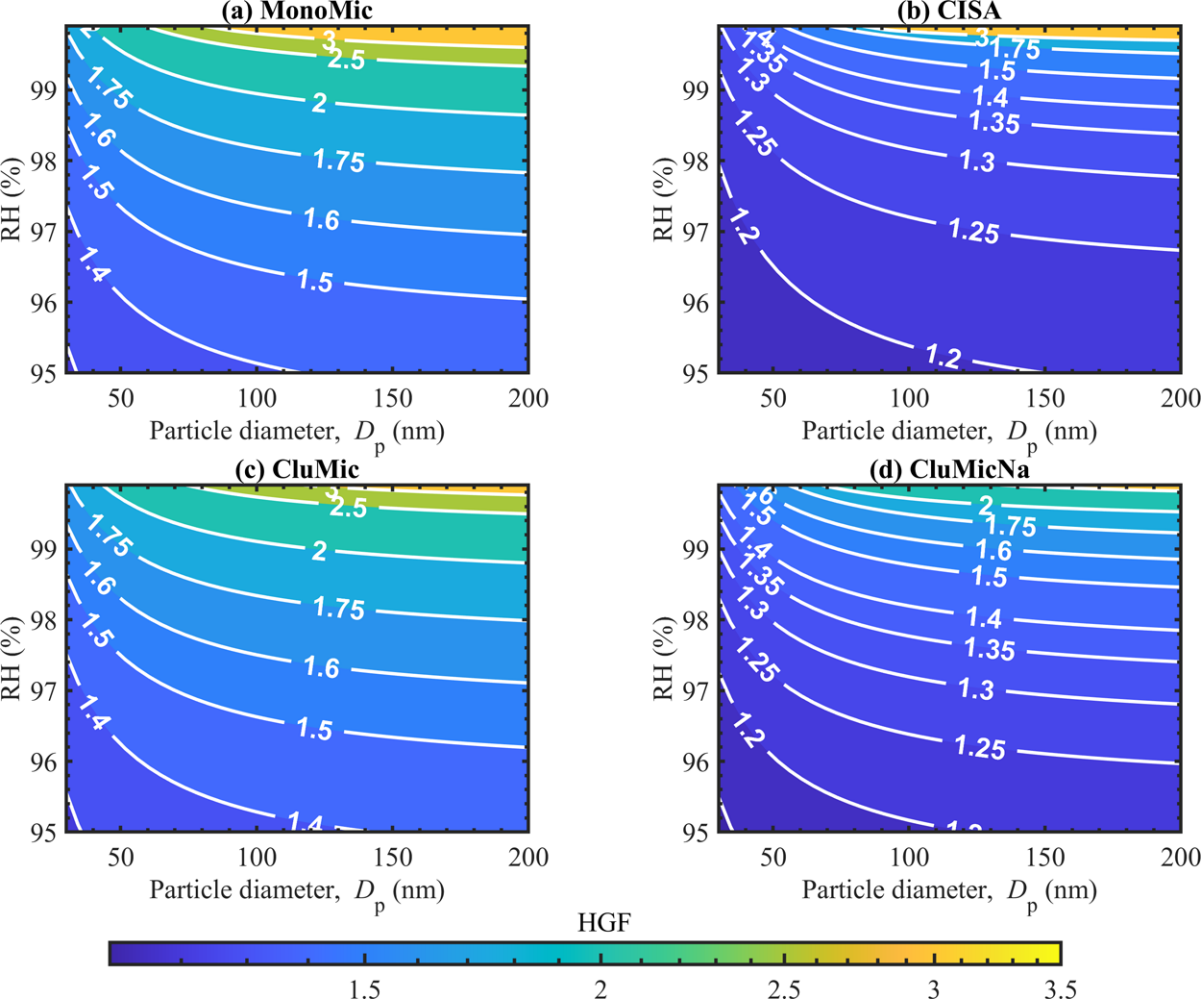
Hygroscopic growth factors (HGF) calculated with eq 11 using the predictions of the MonoMic (a), CISA (b), CluMic (c), and CluMicNa (d) models for NaC_10_ particles of dry sizes *D*_p_ = 30–200 nm at RH = 95–99.9%.

**Figure 5:**
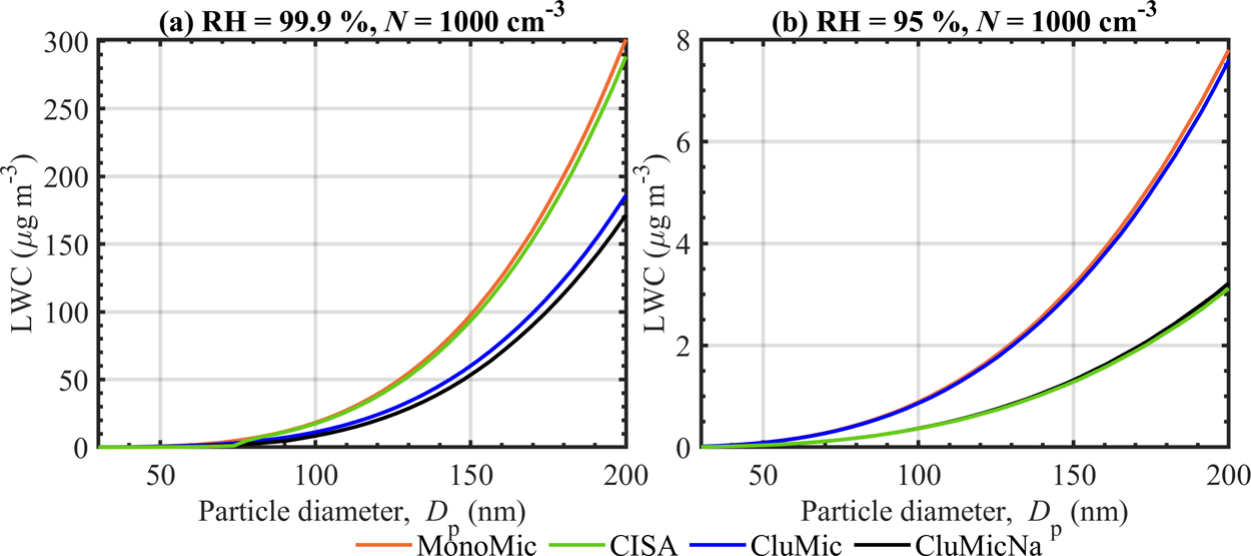
Aerosol liquid water content (LWC) in a population with a fixed droplet concentration *N* = 1000 cm^–3^, calculated for NaC_10_ particles with *D*_p_ = 30–200 nm at RH = 99.9% (a) and 95% (b), using predictions of the MonoMic, CISA, CluMic, and CluMicNa water activity models in eq 12.

#### 3.3.1 Hygroscopic Growth Factors

Figure 4 shows that the effects of various surfactant self-assembly phenomena on water uptake are highly sensitive to RH. This is due to the large influence of self-assembly on on water activity for RH = 95–99.9% (Figure 3). The predicted HGF increases with increasing RH for all water activity models. The CluMic and MonoMic models predict similar HGF across the humidity and particle size ranges, although the CluMic model predicts slightly lower HGF. At RH ∼ 99.9%, HGFs for the CluMic model are significantly lower (Figure S2 of the Supplement), showing the effect of small clusters on the HGF near the maximum of the growth curve before activation (Figure 2). The CISA and CluMicNa models also predict mutually similar HGF. The CluMicNa model predicts slightly larger HGF than CISA, as *a*_w_ > 0.95 where the NaC_10_ concentration exceeds CMC correspond to smaller droplet sizes for the CISA model (Figure 3). At RH ∼ 99.9%, the CISA model however predicts larger HGF than the CluMicNa model. From Figure S2 of the Supplement, we see that for initially dry particles with *D*_p_ ∼ 74–82 nm, at RH = 99.9% droplets have grown to become sufficiently dilute that the surfactant bulk concentration is below the CMC. As no micelles are present in the droplets, the effect of counterion binding vanishes. As a result, the HGF predicted with the CISA model increase to similar values as for the MonoMic model at larger particle sizes. This is illustrated in Figure 1 as the transition from region **(1)** to region **(2)** on the Köhler curve. In Figure 2b, an example of this transition is also seen for particles with *D*_p_ = 80 nm, in case of the CISA model around RH = 99.9%.

Comparing the HGF predicted with the CISA and CluMicNa models to those with MonoMic and CluMic shows that counterion binding to the micelles significantly reduces the particle water uptake for RH ≲ 99%. At these humidities, the surfactant concentration in the droplet bulk exceeds the CMC for the entire range of investigated *D*_p_. The range of HGF predicted with the four models for *D*_p_ = 200 nm at RH = 95% span HGF_MonoMic_ = 1.42 (no counterion binding) and HGF_CISA_ = 1.20, corresponding to a decrease of |HGF_CISA_ – HGF_MonoMic_|/HGF_MonoMic_ ∼ 15%. This translates into a decrease in equilibrium droplet volume of |*V*_CISA_ − *V*_MonoMic_|/*V*_MonoMic_ ∼ 39%, with *V*_MonoMic_ = $\frac{\pi }{6}d^{3}$ and *V*_CISA_ = $\frac{\pi }{6}(0.85d{)}^{3}$ ≈ $\frac{\pi }{6}0.61d^{3}.$

#### 3.3.2 Liquid Water Content

Figure 5 shows that the LWC predicted for an aerosol population with the different water activity models increase significantly with increasing RH and dry particle size *D*_p_. At RH = 99.9% (Figure 5a), the maximum LWC, seen for the largest dry particle size *D*_p_ = 200 nm, are 301 μg m^–3^ (MonoMic), 289 μg m^–3^ (CISA), 186 μg m^–3^ (CluMic), and 172 μg m^–3^ (CluMicNa). The difference between the maximum LWC for the MonoMic and CISA models is ∼4%, calculated as (LWC_CISA_ – LWC_MonoMic_)/LWC_MonoMic_ from unrounded maximum LWC values and then rounding to 1% accuracy. For *D*_p_ = 200 nm, micelles are not present at droplet states corresponding to RH = 99.9% (Figures 1 and 2) and differences in LWC can be attributed to nonideal interactions in the droplet solution which are accounted for with CISA, but not the MonoMic model. Between the MonoMic and CluMic models, there is a ∼38% decrease in the predicted LWC at *D*_p_ = 200 nm, caused by the reduced HGF due to the formation of small clusters predicted with the CluMic model (Figure S2). Similarly, there is a ∼43% decrease in LWC between MonoMic and CluMicNa due to the combined effects of small clusters and counterion binding to micelles in the latter model.

At RH = 95% (Figure 5b), the maximum LWC is 8 μg m^–3^ for the MonoMic and CluMic models and 3 μg m^–3^ for the CISA and CluMicNa models. CISA and CluMicNa predict relative decreases in the LWC of ∼60 and 59%, respectively, compared to the MonoMic model. Figure S1 at RH = 95% shows that the CISA and CluMicNa models predict higher NaC_10_ droplet bulk concentrations compared to MonoMic and CluMic. The total amount of solute in a growing droplet is constant and the bulk–surface partitioning is identical between the water activity models at a given droplet size. The NaC_10_ bulk concentration is therefore the same between different models at each droplet size and higher NaC_10_ concentrations imply that the water content is lower (Figure 1). The differences between LWC at RH = 99.9 and 95% show that for lower humidities, where micelles are present in the droplet bulk, counterion binding to the micelles leads to a significant decrease in the LWC, analogously to the HGF (Figure S2). The LWC predicted with the MonoMic and CluMic models are significantly different at RH = 99.9% (∼38%) but similar at 95%, showing that the relative impact of small surfactant clusters in the droplets on the LWC decreases together with the RH.

Nguyen et al.^64^ estimated the average aerosol LWC for urban, urban downwind, and rural areas to be 14, 15, and 3.2 μg m^–3^, suggesting that water uptake by inorganic compounds dominates over contributions by organic compounds. They also estimated that the highest LWC concentration of the sites studied was Beijing with 86 μg m^–3^ and the lowest was Chebogue with 0.42 μg m^–3^. Jin et al.^65^ found that organic aerosol components could have a significant contribution to atmospheric aerosol LWC in Beijing (∼30 ± 22%). They also reported that during seven heavy pollution events, aerosol LWC on average increases from 8 to 89 μg m^–3^ as ambient RH increases from 15 to 80%. Su et al.^66^ estimated the LWC of PM_2.5_ over four seasons with the ISORROPIA II thermodynamic model. The seasonal average showed that LWC increased from ∼3 to ∼76 μg m^–3^ when ambient RH increased from 30 to 90% for Beijing.

The humidity conditions for LWC predictions in Figure 5 are higher than those investigated by Jin et al.^65^ and Su et al.^66^ and the aerosol number concentration *N* = 1000 cm^–3^ is at the lower end of the range for remote continental aerosol.^67^ Droplet concentrations could be several orders of magnitude higher in polluted urban areas, such as Beijing. In this work, we estimate the potential impact on the aerosol LWC specifically from NaC_10_, representing common atmospheric surfactants present in many environments,^16^ including urban sites such as Beijing.^68^ The differences in predicted LWC between the different water activity models employed are of comparable magnitude to LWC commonly observed in atmospheric aerosols.^64–66^ This suggests that accounting for surfactant self-assembly phenomena could affect predictions of ambient LWC in regions where ionic surfactants contribute significantly to aerosol mass. Especially in conditions where micelles are present in the droplets, counterion binding to the micelles could strongly decrease the LWC.

Overall, our results show that surfactant self-assembly phenomena in the droplet bulk phase can have significant implications for subsaturated aerosol hygroscopic growth and LWC, but only minor effects on droplet activation. If no consideration was given to self-assembly phenomena, including the CMC itself, and the surfactant was treated as a regular, soluble solute, the increased amount of dissociated solute entities would lead to predicting lower droplet water activities (Figure 3) than for the models presented here. This would result in correspondingly larger equilibrium droplet sizes (Figure 4) and higher liquid water content (Figure 5) at a given RH when surfactant concentrations exceed the CMC. Aerosol size and LWC directly impact atmospheric visibility and aerosol optical properties,^69^ as well as the radiative properties of aerosol.^70,71^ Water content could also affect the concentrations of other partially soluble aerosol components by governing aqueous phase partitioning from both gas and condensed phases,^72,73^ which may in turn impact aqueous phase chemistry.

The significant effects of surfactant self-assembly on aqueous droplet growth seen in this work underscore the importance of both theoretical model development and experimental validation to firmly establish the impact for atmospheric aerosols. However, the underlying processes related to surfactant self-assembly are expected to affect complex droplets in the atmosphere via similar mechanisms as the phenomena investigated here.

## Supplementary Material



## Data Availability

Data underlying the figures are available through the Zenodo online data repository: https://doi.org/10.5281/zenodo.15647532.

## Notes

Description of bulk phase sodium decanoate concentration calculations (Section S1.1) and figures showing calculated results (Figure S1) and additional hygroscopic growth factor and liquid water content for RH = 95–99.9% (Section S1.2 and Figure S2); description of the CISA activity model calculations (Section S2.1) and table of model parameters (Table S1); parametrizations and parameter values for the binary surface tension (Section S3.1); solution density (Section S3.2); CMC (Section S3.3); degree of micellization (Section S3.4); and sodium decanoate experimental critical supersaturation values (Section S3.5) (PDF)
