# Supplementary material for: Impact of Bulk-Phase Self-Assembly on Growth and Activation of Aqueous Surfactant Aerosol
Source: Environ Sci Technol. 2025 Jul 10;59(28):14552–63. doi: 10.1021/acs.est.4c11584 (PMC12288086; doi:10.1021/acs.est.4c11584)
Supplement: Supplementary file 1 [file es4c11584_si_001.pdf]

Supporting Information for: Impact of Bulk-Phase Self-Assembly on Growth and Activation of Aqueous Surfactant Aerosol

Sampo Vepsäläinen[†,‡] and Nønne L. Prisle[*,‡,¶,§]

†*Nano and Molecular Systems Research Unit, University of Oulu, P.O. Box 3000, FI-90014, Oulu, Finland*

‡*Center for Atmospheric Research, University of Oulu, P.O. Box 4500, FI-90014, Oulu, Finland*

¶*Center for Molecular Water Science, Deutsches Elektronen-Synchrotron DESY, Notkestrasse 85, D-22607 Hamburg, Germany*

§*Institute of Inorganic and Applied Chemistry, University of Hamburg, Martin-Luther-King-Platz 6, D-20146 Hamburg, Germany*

E-mail: nonne.prisle@oulu.fi

Number of pages: 10

Number of figures: 2

Number of tables: 1

S1 Additional results

S1.1 Bulk phase sodium decanoate concentrations

Figure S1 shows the bulk phase NaC_{10} molar concentrations predicted with the MonoMic, CISA, CluMic and CluMicNa water activity models for RH $\approx 95 - 99.9$ % and dry particle sizes $D_\mathrm{p} = 30 - 200$ nm. The bulk phase NaC_{10} molar concentrations have been calculated from the corresponding mole fractions (x_j^B for j = w and NaC_{10}) given by the Monolayer model as

$$c_{\mathrm{NaC}_{10}}^{\mathrm{B}} = \frac{\rho(\mathrm{x}^\mathrm{B})}{M_\mathrm{w} x_\mathrm{w}^\mathrm{B} + M_{\mathrm{NaC}_{10}} x_{\mathrm{NaC}_{10}}^\mathrm{B}} x_{\mathrm{NaC}_{10}}^\mathrm{B}, \tag{S1}$$

where $\rho(\mathrm{x}^\mathrm{B})$ is the solution density (kg m^{-3}, Section S3.2), and M_j is the molar mass for j = w and NaC_{10} (kg mol^{-1}).

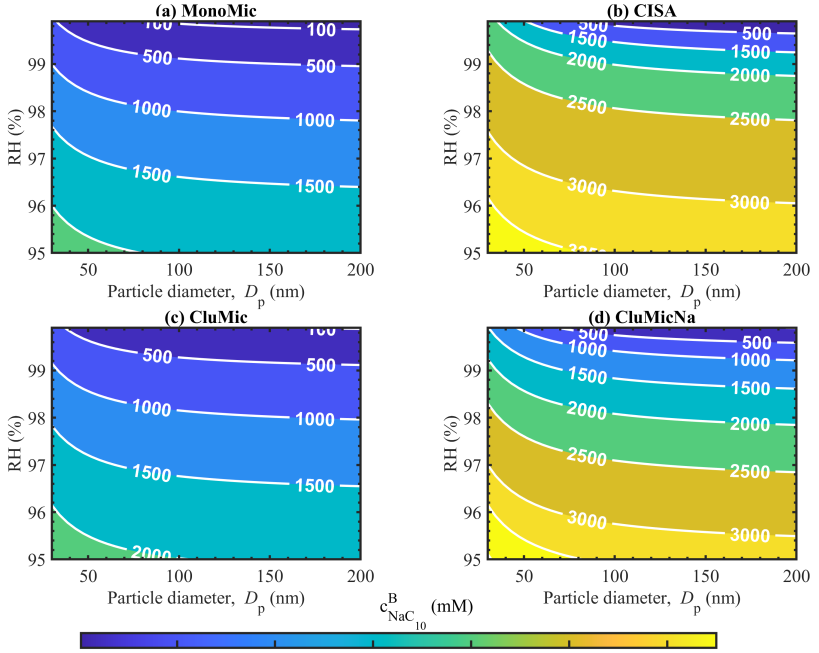

Figure S1: Bulk phase NaC_{10} molar concentrations $c_{\mathrm{NaC}_{10}}^{\mathrm{B}}$ for the MonoMic, CISA, CluMic and CluMicNa water activity models in panels (a), (b), (c), and (d). The concentrations are shown between RH $\approx 95 - 99.9$% for dry particle sizes $D_\mathrm{p} = 30 - 200$ nm.

S1.2 HGF and LWC additional figures

In Figure S2, we present the hygroscopic growth factors (HGF) in panels (a), (c), (e), (g), (i), and (k) and the aerosol liquid water content (LWC) in panels (b), (d), (f), (h), (j), and (l) at RH = 99.9 − 95 % for NaC_{10} particles with $D_p = 30 - 200$ nm.

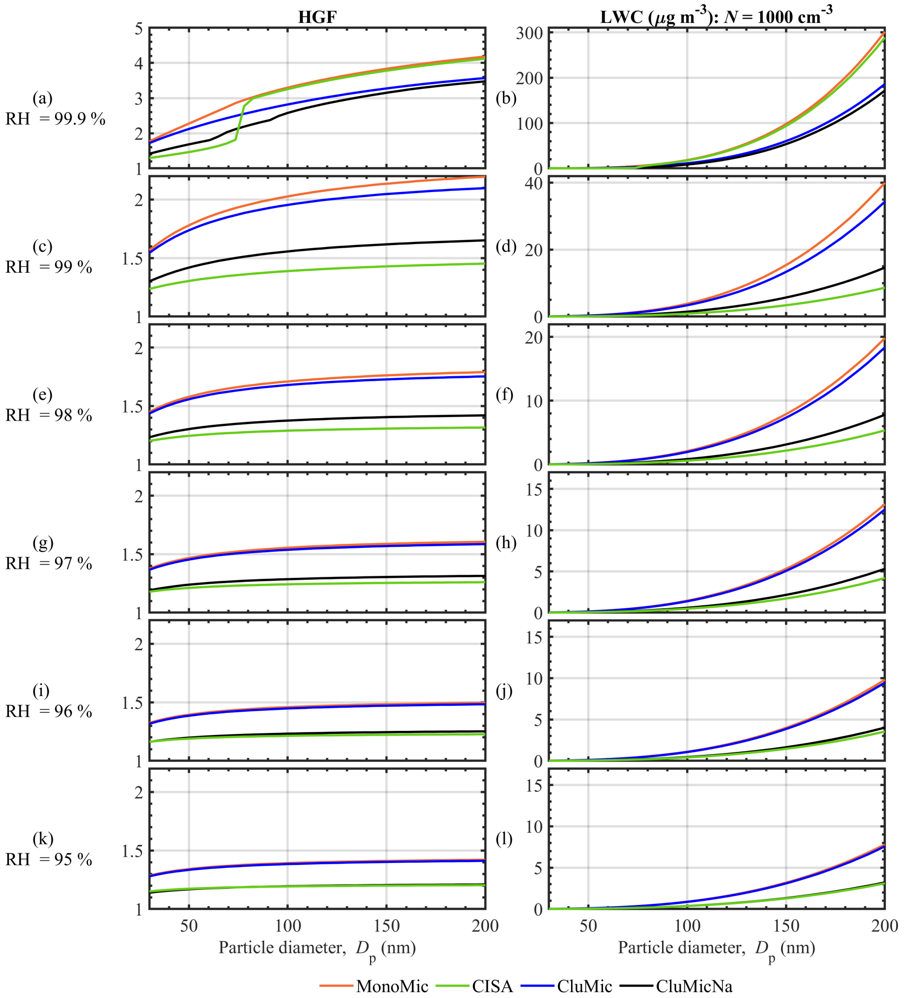

Figure S2: Panels (a), (c), (e), (g), (i), and (k) show the hygroscopic growth factors (HGF) calculated from the predictions of the MonoMic, CISA, CluMic, and CluMicNa models for particles ($D_p = 30 - 200$ nm) of NaC_{10} at RH = 99.9 %, 99 %, 98 %, 97 %, 96 % and 95 %. Panels (b), (d), (f), (h), (j), and (l) display the aerosol liquid water content (LWC) calculated for a constant droplet number concentration of $N = 1000$ cm^{-3}.

S2 Calculation details

S2.1 CISA activity model

In the CISA model,[1] a surfactant MX forms micelles above the CMC according to the reaction

$$qM^+ \quad + \quad n_m X^- \quad \rightleftharpoons \quad (M_q X_{n_m})^{q-n_m} \quad , \tag{S2}$$

where $(M_q X_{n_m})^{q-n_m}$ represents the micelle (mic). Here we consider micelles with $n_\mathrm{m} = 48$, where the number of counterions bound to each micelle is $q = 0.68 n_\mathrm{m}$.[2,3] The activity of water is calculated as

$$a_\mathrm{w} = \exp\left(-\frac{\phi^\mathrm{ion}}{s}\left(m_\mathrm{M} + m_\mathrm{X} + m_\mathrm{mic}\right)\right) \tag{S3}$$

where ϕ^ion is the osmotic coefficient, s is the molality of pure water, m_M and m_X are the molalities of the sodium and decanoate ions, respectively, in solution and m_mic represents the molality of micelles. The reference state of each ion is a hypothetical 1 molal solution, for which the ratio $a_i m_i^{-1}$ tends to unity as the molality approaches zero.[4] The osmotic coefficient is given as

$$\phi^\mathrm{ion} = 1 + \frac{2}{\sum m_i}\left(-\frac{A^\phi I^{3/2}}{1 + b\sqrt{I}} + m_\mathrm{M}\left(m_\mathrm{X}\left(B^\phi_\mathrm{MX} + EC^\phi_\mathrm{MX}\right) + m_\mathrm{mic}\left(B^\phi_\mathrm{Mmic} + EC^\phi_\mathrm{Mmic}\right)\right)\right), \tag{S4}$$

where b is a model constant equal to 1.2 kg$^{0.5}$ mol$^{-0.5}$ and I is the ionic strength calculated as

$$I = 0.5\left(m_\mathrm{M} + m_\mathrm{X} + \eta^2 (q - n_m)^2 m_\mathrm{mic}\right), \tag{S5}$$

where $\eta = 0.5$ is shielding factor to reduce the effect of the micellar charge on the ionic strength of the solution. The equivalent electrical molality E is calculated as

$$E = m_{\mathrm{X}} + \eta(n_m - q)m_{\mathrm{mic}}. \tag{S6}$$

The model parameters of the form B_{ca} and C_{ca} where "c" and "a" stand for cation and anion, respectively, account for pair and triple interactions between $Na^+$and $C_{10}{}^-$ ions and Na^+ions and micelles. The parameter B^{ϕ}_{ca} is calculated as

$$B^{\phi}_{\mathrm{ca}} = \beta^{(0)}_{\mathrm{ca}} + \beta^{(1)}_{\mathrm{ca}} \exp\left(-\alpha_1\sqrt{I}\right) \tag{S7}$$

where α_1 is a model parameter equal to 2.0 $\mathrm{kg}^{0.5}$ $\mathrm{mol}^{-0.5}$ for all symmetric electrolytes. The corresponding values of model parameters C^{ϕ}_{ca} and β_{ca} are shown in Table S1. The Debye-Hückel limiting slope $A^{\phi} = A^{\gamma}/3$ is given as

$$A^{\gamma} = (2\pi N_{\mathrm{A}}\rho_{\mathrm{w}})^{\frac{1}{2}} \left(\frac{e^2}{4\pi\varepsilon_0 D k_{\mathrm{B}} T}\right)^{\frac{3}{2}} \tag{S8}$$

where k_{B} is the Boltzmann constant, N_{A} the Avogadro's number, T the temperature, ρ_{w} the density of water[5], e the electron charge, D the dielectric constant of water[6] and ε_0 the permittivity of vacuum.

Table S1: The CISA model parameters to calculate the activity coefficients in aqueous solutions of NaC_{10}. M, X and mic refer to sodium ion, decanoate ion, and micelle, respectively. Parameter $\beta^{(0)}_{\mathrm{ca}}$ and $\beta^{(1)}_{\mathrm{ca}}$ values for eq. S7 are listed in the first and second row, respectively.[1]

Solute	β_{MX} (kg mol^{-1})	C^{ϕ}_{MX} (kg^2 mol^{-2})	β_{Mmic} (kg mol^{-1})	C^{ϕ}_{Mmic} (kg^2 mol^{-2})
NaC_{10}	−0.9259	4.6700	1.1596	−0.6462
	1.7760		8.1080	

S3 Solution and compound properties

S3.1 Binary surface tension

We used the Langmuir-Szyszkowski equation with a parameterization scheme similar to Prisle et al.[7] to calculate the surface tension of the aqueous surfactant solutions as

$$\sigma = \sigma_{\mathrm{w}}(T) - a \ln\left(1 + \frac{m_{\mathrm{NaC_{10}}}}{b}\right), \quad \text{(S9)}$$

where $\sigma_{\mathrm{w}}(T)$ is the surface tension of pure water as a function of temperature (T)[8], $m_{\mathrm{NaC_{10}}}$ is the molality of $\mathrm{NaC_{10}}$, and $a = 10.1337$ mN m^{-1} and $b = 8.696 \cdot 10^{-4}$ mol kg^{-1} are the model parameters fitted to experimental surface tension data at 296.15 K.[9] Equation S9 is assumed to scale with $\sigma_{\mathrm{w}}(T)$ and used here with $\sigma_{\mathrm{w}}(T = 298.15\ \mathrm{K}) = 72.0$ mN m^{-1} (Table 1 of the main paper). The droplet surface tension σ is constrained at a lowest possible value value of 22.5 mN m^{-1}, corresponding to the surface tension at the CMC (σ_{CMC}) as given by eq. S9 at the CMC value of 0.1136 mol kg^{-1}.[1] The surface tension of the pure surfactant is assumed equal to σ_{CMC}.

S3.2 Binary solution density

The density of the binary water–$\mathrm{NaC_{10}}$ solution is a composition-dependent function calculated with the parametrization of Calderón and Prisle[10]. For more information about the density function employed in the current work, we refer to the original publication[10] that contains extensive documentation.

The density of aqueous surfactant solutions is calculated using the apparent molal volume of the surfactant $\Phi_{\mathrm{NaC_{10}}}$ as

$$\rho\left(T, m_{\mathrm{NaC_{10}}}\right) = \frac{1 + m_{\mathrm{NaC_{10}}} M_{\mathrm{NaC_{10}}}}{1/\rho_{\mathrm{w}} + 1 \cdot 10^{-6} \Phi_{\mathrm{NaC_{10}}} m_{\mathrm{NaC_{10}}}} \quad \text{(S10)}$$

where $m_{\mathrm{NaC_{10}}}$ and $M_{\mathrm{NaC_{10}}}$ are the molality and molar mass of the surfactant, respectively, and ρ_{w} is the density of pure water (Table 1 of the main article). The apparent partial molal volume of the surfactant changes due to micellization and its value can be calculated as

$$\Phi_{\mathrm{NaC_{10}}} = H\left(\mathrm{CMC} - m_{\mathrm{NaC_{10}}}\right)\left(\Phi^{\infty}_{\mathrm{NaC_{10}}} + A_v\sqrt{m_{\mathrm{NaC_{10}}}} + B^{V}_{\mathrm{NaC_{10}}} m_{\mathrm{NaC_{10}}}\right) + H\left(m_{\mathrm{NaC_{10}}} - \mathrm{CMC}\right)\left((1-\xi)\Phi_{\mathrm{NaC_{10},CMC}} + \xi V_{\mathrm{mic}}\right), \quad \text{(S11)}$$

where CMC is in molal units, $\Phi^{\infty}_{\mathrm{NaC_{10}}}$ and $\Phi_{\mathrm{NaC_{10},CMC}}$ are the apparent molal volume of the surfactant at infinite dilution and at the CMC in cm^{3}mol^{-1}, respectively, H is the Heaviside step function and ξ is the degree of micellization (Section S3.4).

The model parameter $B^{V}_{\mathrm{NaC_{10}}}$ is used to include the effect of surfactant–surfactant interactions with consistent units of cm^3 mol^{-2} kg. These model parameters can be obtained from their temperature-dependent parameterizations as

$$\Phi^{\infty}_{\mathrm{NaC_{10}}} = 0.2762T + 82.06 \quad \text{(S12)}$$

$$\Phi_{\mathrm{NaC_{10},CMC}} = 0.3004T + 75.65 \quad \text{(S13)}$$

and

$$B^{V}_{\mathrm{NaC_{10}}} = 0.002785T^2 - 1.648T + 244.5. \quad \text{(S14)}$$

The variable V_{mic} is the surfactant molal volume in micellar form. Its value depends on the change in molar volume experienced by the surfactant during micellization as

$$V_{\mathrm{mic}} = \Phi_{\mathrm{NaC_{10},CMC}} + \Delta V_{\mathrm{mic}}. \quad \text{(S15)}$$

The ΔV_{mic} is a temperature-dependent parameter that can be calculated in units of cm^3 mol^{-1}

as

$$\Delta V_{\mathrm{mic}} = -0.1107T + 44.5 \tag{S16}$$

The variable A_v represents the Debye-Hückel limiting slope in volumetric units $\left(\mathrm{cm}^3\ \mathrm{kg}^{1/2}\ \mathrm{mol}^{-3/2}\right)$, and is calculated as

$$\begin{aligned} A_v =& 1.50619 + 0.0130073(T - 273.15) + 4.8307 \cdot 10^{-5}(T - 273.15)^2 + 8.95087 \cdot 10^{-7}(T - 273.15)^3 \\ & - 3.727 \cdot 10^{-9}(T - 273.15)^4 + 2.3942 \cdot 10^{-11}(T - 273.15)^5. \end{aligned} \tag{S17}$$

S3.3 Critical Micelle Concentration

The CMC used for $\mathrm{NaC_{10}}$ at 298.15 K is 0.1136 mol kg^{-1} in terms of molality.[1] The corresponding mole fraction is estimated to be $x_{\mathrm{CMC}} = 0.002043$, and the molar concentration $\sim$ 111 mM.

S3.4 Degree of micellization

The extent, or degree, of micellization in the MonoMic, CluMic, CISA, and CluMicNa water activity models is quantified by the variable ξ using the CMC in molality as

$$\xi = \left(\frac{(\mathrm{sgn}\,(m_{\mathrm{NaC_{10}}} - \mathrm{CMC}) + 1)}{2}\right) \frac{m_{\mathrm{NaC_{10}}} - \mathrm{CMC}}{m_{\mathrm{NaC_{10}}}} \tag{S18}$$

where sgn is the sign function.[1,10,11] The variable ξ determines the fraction of the surfactant monomers left in the droplet bulk, which form micelles above the CMC

$$n_{\mathrm{mic}}^{\mathrm{B}} = \xi \frac{n_{\mathrm{NaC_{10}}}}{n_m}, \tag{S19}$$

where $n_{\mathrm{NaC_{10}}}$ is the amount of sodium decanoate left in the droplet bulk after bulk–surface partitioning. Correspondingly, $(1 - \xi)$ is the fraction that either remains as monomers or

forms clusters, depending on the water activity model.

S3.5 Experimental critical supersaturations for sodium decanoate

Prisle et al.[7] measured experimental critical supersaturations and fitted the results as power functions dependent on the dry particle diameter. The equation given for NaC_{10} is

$$y = 257.68 \cdot x^{-1.4814} \tag{S20}$$

where y denotes the SS_c in % and x the dry particle size in nm.

References

(1) Calderón, S. M.; Malila, J.; Prisle, N. L. Model for estimating activity coefficients in binary and ternary ionic surfactant solutions. *J. Atmos. Chem.* **2020**, *77*, 141–168, DOI: 10.1007/s10874-020-09407-4.

(2) Vikingstad, E.; Skauge, A.; Høiland, H. Partial molal volumes and compressibilities of the homologous series of sodium alkylcarboxylates, R6COONa–R13COONa, in aqueous solution. *J. Colloid Interface Sci.* **1978**, *66*, 240–246, DOI: 10.1016/0021-9797(78)90301-6.

(3) Burchfield, T. E.; Woolley, E. M. Model for thermodynamics of ionic surfactant solutions. 1. Osmotic and activity coefficients. *J. Phys. Chem.* **1984**, *88*, 2149–2155, DOI: 10.1021/j150654a042.

(4) Pitzer, K. *Activity Coefficients in Electrolyte Solutions*; CRC Press, 2017.

(5) Pátek, J.; Hrubý, J.; Klomfar, J.; Součková, M.; Harvey, A. H. Reference Correlations for Thermophysical Properties of Liquid Water at 0.1 MPa. *J. Phys. Chem. Ref. Data* **2009**, *38*, 21–29, DOI: 10.1063/1.3043575.

(6) Fernández, D. P.; Goodwin, A. R. H.; Lemmon, E. W.; Levelt Sengers, J. M. H.; Williams, R. C. A Formulation for the Static Permittivity of Water and Steam at Temperatures from 238 K to 873 K at Pressures up to 1200 MPa, Including Derivatives and Debye–Hückel Coefficients. *J. Phys. Chem. Ref. Data* **1997**, *26*, 1125–1166, DOI: 10.1063/1.555997.

(7) Prisle, N. L.; Raatikainen, T.; Sorjamaa, R.; Svenningsson, B.; Laaksonen, A.; Bilde, M. Surfactant partitioning in cloud droplet activation: a study of C8, C10, C12 and C14 normal fatty acid sodium salts. *Tellus B* **2008**, *60*, 416–431, DOI: 10.1111/j.1600-0889.2008.00352.x.

(8) International Association for the Properties of Water and Steam (IAPWS) Revised Release on Surface Tension of Ordinary Water Substance: IAPWS R1-76 (2014). *Moscow* **2014**, last access: 18.3.2020.

(9) Prisle, N. Surface tension of aqueous sodium decanoate measured with Wilhelmy plate tensiometry. 2025; https://doi.org/10.5281/zenodo.15085896.

(10) Calderón, S. M.; Prisle, N. L. Composition dependent density of ternary aqueous solutions of ionic surfactants and salts. *J. Atmos. Chem* **2021**, *78*, 99–123, DOI: 10.1007/s10874-020-09411-8.

(11) Lisi, R. D.; Perron, G.; Desnoyers, J. E. Volumetric and thermochemical properties of ionic surfactants: sodium decanoate and octylamine hydrobromide in water. *Can. J. Chem.* **1980**, *58*, 959–969, DOI: 10.1139/v80-152.
